# The prevalence of familial hypercholesterolemia in the West Siberian region of the Russian Federation: A substudy of the ESSE-RF

**DOI:** 10.1371/journal.pone.0181148

**Published:** 2017-07-18

**Authors:** Alexandra I. Ershova, Alexey N. Meshkov, Stepan S. Bazhan, Marina A. Storozhok, Alexey Y. Efanov, Irina V. Medvedeva, Elena V. Indukaeva, Yana V. Danilchenko, Olga K. Kuzmina, Olga L. Barbarash, Alexander D. Deev, Svetlana A. Shalnova, Sergey A. Boytsov

**Affiliations:** 1 National Research Center for Preventive Medicine, Moscow, Russia; 2 Institution of Internal and Preventive Medicine, Novosibirsk, Russia; 3 Tyumen State Medical Academy, Tyumen, Russia; 4 Research Institute for Complex Issues of Cardiovascular Diseases, Kemerovo, Russia; McMaster University, CANADA

## Abstract

**Background:**

The prevalence of familial hypercholesterolemia (FH) in Russia has not previously been evaluated. The aim of our study was to investigate the prevalence of FH in the population of the West Siberian region of Russia, and then estimate the frequency of coronary artery disease (CAD) and treatment with cholesterol-lowering medication in FH patients.

**Methods:**

The sample of our study consisted of participants from the population-based cohort of The Epidemiology of Cardiovascular Risk Factors and Diseases in Regions of the Russian Federation Study (ESSE-RF), conducted in the Tyumen and Kemerovo regions (1,630 and 1,622 people, respectively, aged 25–64). All participants who had LDL-cholesterol higher than 4.9 mmol/l and who had LDL-cholesterol less than or equal to 4.9 mmol/l but had statin therapy were examined and interviewed by experts in FH.

**Results:**

The prevalence of patients with definite FH was 0.24% (one in 407) (95% confidence interval [CI]: 0.06%–0.42%), with probable FH was 0.68% (one in 148) (95% CI: 0.38%–0.98%), and with definite or probable FH combined was 0.92% (one in 108) (95% CI: 0.58%–1.26%). 40% (95% CI: 20.8%–59.2%) of patients with definite or probable FH had CAD. However, only 23% (95% CI: 6.3%–39.7%) of patients with definite or probable FH were on statins. The odds ratios for CAD and myocardial infarction (MI), adjusted for age, gender, region, smoking, hypertension, and diabetes mellitus, were 3.71 (95% CI: 1.58–8.72) (*p* = 0.003) and 4.06 (95% CI: 0.89–18.55) (*р* = 0.070) respectively for individuals with definite or probable FH relative to those who were unlikely to have FH.

**Conclusions:**

The prevalence of FH in Russia may be significantly higher than previously estimated. There is underdiagnosis and undertreatment of FH in Russia.

## Introduction

Familial hypercholesterolemia (FH) is an autosomal dominant disorder known to be associated with elevated cholesterol levels and an increased risk of premature coronary artery disease. Historically, the community prevalence of heterozygous FH is estimated to be one in 500 [1]. This estimate is imprecise, and is based on calculations using the Hardy-Weinberg equation p^2^+2pq+q^2^ = 1, with q^2^ being the observed frequency of FH homozygotes in a country and p and q being the frequency of the normal and FH causing alleles, respectively [2]. The prevalence is known to be higher in countries where a founder gene effect has occurred. For instance, the heterozygous carrier frequency was estimated to be one in 270 in the French Canadian population of the Quebec province [3] and even as high as one in 70 in Afrikaners in South Africa [4].

Recent data suggests that the real prevalence of FH is underestimated. As a result of improvements in molecular genetics methods, the frequency of molecularly homozygous FH was investigated in an open society—the Netherlands [5]. It was shown that the prevalence of molecularly defined homozygous FH was much higher than previously assumed and was estimated to be about one in 300,000. Therefore, the prevalence of heterozygous FH may be about one in 200. The Copenhagen General Population Study was the first unselected, community-based population study that assessed the prevalence of FH [6]. It was determined that the prevalence in individuals classified with definite or probable FH approached one in 137, and at least half of affected subjects were not receiving cholesterol-lowering medication. However, a corrigendum in the study was later published and FH prevalence in the corrected version was 1:223 [7].

The prevalence of FH in the West Siberian region of Russia had not previously been evaluated. The aim of our study was to investigate the prevalence of FH in the Russian population. Additionally, we assessed the prevalence of CAD and the treatment with cholesterol-lowering medication in FH patients.

## Materials and methods

### Sampling

Individuals for our study were selected from the Epidemiology of Cardiovascular Risk Factors and Diseases in Regions of the Russian Federation Study (ESSE-RF). The ESSE-RF is a multicentre population-based study, conducted from 2012 to 2013, covering 13 regions of Russia, differing in climatic, geographic, economic, and demographic characteristics [8]. About 2,000 people, aged 25–64, from every region were randomly selected for the study. The multi-stage clustered sample was obtained using Kish methods [9].

District outpatient departments (polyclinics) were randomly selected as primary sampling units (PSU), each from 30,000 to 80,000 adult inhabitants. Then, five sites (physician’s localities) from every polyclinic were randomly selected as a secondary sampling unit (SSU), which consisted of approximately 2000 adults. On each site, 100 households were selected as tertiary sampling units (TSU) and in every household an available study subject was found. One PSU in selected regions was rural while all the others were urban. The total sample size was calculated using the formula: 4PSU*5SSU*100TSU = 2000 subjects, selected to be observed in the region with intended response rate of 80%. Data was obtained from questionnaires administered face-to-face and from fasting venous blood samples. The level of low-density lipoprotein cholesterol (LDL-cholesterol) was measured directly in all participants. All analyses were done in a high-quality central laboratory using the ARCHITECT c8000 clinical chemistry analyzer and diagnostic kits (Abbott). All subjects were interviewed to assess statin treatment. CAD and prior MI were defined either by the Rose Angina Questionnaire or the patients’ positive answer to questions concerning previous diagnoses of either coronary artery disease or myocardial infarction.

**Fig 1:**
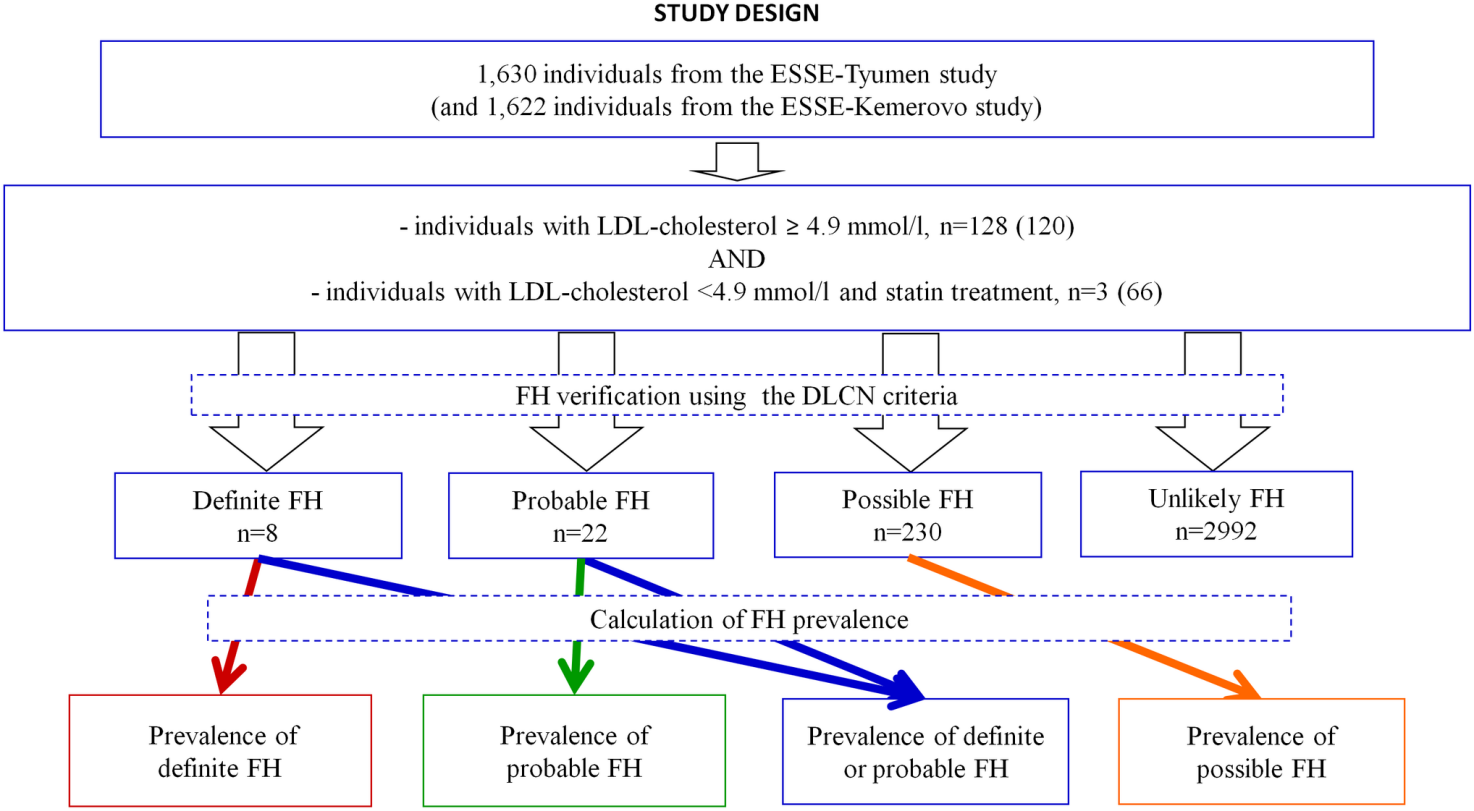
Study design.

The sample of our study consisted of all participants of the ESSE-RF, conducted in the Tyumen and Kemerovo regions. Regions for assessment of FH prevalence in Russia were selected randomly. The samples from the Tyumen and Kemerovo regions (ESSE-Tyumen and ESSE-Kemerovo studies) included 1,630 and 1,622 people, respectively, aged 25–64. In the Tyumen and Kemerovo regions, respectively, 30% and 43% were men. All participants who had LDL-cholesterol higher than 4.9 mmol/l (142 and 138 persons from Tyumen and Kemerovo regions, respectively) and who had LDL-cholesterol less than or equal to 4.9 mmol/l but had statin therapy (10 and 71 persons from Tyumen and Kemerovo regions, respectively) were examined and interviewed by experts in FH additionally (Fig 1). We assume that the higher frequency of statin prescription in the Kemerovo region is probably due to differences in the public health systems of the two regions and, as a result, there is a higher level of cardiological care in the Kemerovo region.

The study was approved by the Independent Ethic Committee of the National Research Center for Preventive Medicine and conducted according to the principles expressed in The Declaration of Helsinki. Informed written consent was obtained from all participants.

### Diagnostic criteria of FH

The diagnosis of FH was determined using the Dutch Lipid Clinical Network Criteria (DLCN) [10]. Criteria taken into account were:

- Family history of premature coronary artery disease (<55 years for men; <60 years for women) in a first-degree relative and/or an increase of LDL-cholesterol more than 4.9 mmol/l in first-degree relatives (1 point)
- Clinical history of premature coronary artery disease (ages as above, 2 points) or premature cerebral or peripheral vascular disease (ages as above, 1 point) in the subject
- Presence of tendon xanthomata in the subject (6 points)
- Presence of corneal arcus in the subject (4 points)
- Level of LDL-cholesterol in the subject higher than 8.5 mmol/l (8 points), 6.5–8.4 mmol/l (5 points), 5.0–6.4 mmol/l (3 points), or 4.0–4.9 mmol/l (1 point).

CAD, MI, cerebral and peripheral vascular diseases were established on the basis of data from participants’ previous medical documentation, which was provided during the visit. For CAD diagnosis, not only diagnosis in the patient's medical records was taken into account, but also the data confirming the diagnosis: a positive stress test, significant stenosis in coronary angiography, prior myocardial infarction, unstable angina, or coronary artery revascularization. MI was confirmed in accordance with the third universal definition of myocardial infarction [11]. Other cholesterol-lowering treatment besides therapy with a statin was not received by any participants. The level of LDL-cholesterol in medical records before starting statin therapy was used to diagnose FH in subjects with statin therapy in the ESSE-RF. Presence of tendon xanthomata and corneal arcus were assessed in every subject during the visit’s physical examination. Relatives data was collected from medical records brought by the participant to the visit or orally obtained. A diagnosis of FH was considered definite if the total score was greater than 8, probable if the score was 6–8, possible if the score was 3–5, and unlikely if the score was below 3 points.

### Statistical analysis

Statistical analysis was made with Statistica 6.0 software. The data below is presented as a median (25th-75th percentile). A *р*-value of less than 0.05 was considered to be statistically significant. The *p*-values for quantitative parameters were calculated using a nonparametric Mann-Whitney test. The *p*-values for quality parameters were calculated using the Yates corrected χ2 test. If a sample size was less than five, the two-tailed Fisher exact test was used.

We calculated the prevalence of FH by dividing the number of people with definite FH, probable FH, definite or probable FH, and possible FH into total sample size consecutively. The prevalence of each FH definition was worked out as a percentage for all participants. Confidence interval was calculated using the formula z_0.95_√(p(1-p)/n)±0.5/n.

Risk of CAD for individuals with a diagnosis of definite/probable and possible FH relative to those with unlikely FH was estimated by multiple logistic regression, adjusting for age, gender, region, smoking, hypertension, and diabetes mellitus, using the SAS software, version 6.12.

## Results

In the Tyumen region, we examined 131 participants (86%) who had a level of LDL-cholesterol higher than 4.9 mmol/l (128 participants) or were undergoing statin treatment (3 participants). In the Kemerovo region we examined 186 participants (89%) who had a level of LDL-cholesterol higher than 4.9 mmol/l (120 participants) or were undergoing statin treatment (66 participants). Clinical characteristics of the subjects are shown in Table 1. There were significant differences in age, sex, and prevalence of ischemic stroke between the two study groups. The prevalence of CAD was higher in the group from the Kemerovo region, but was not significant. However, patients with a level of LDL-cholesterol higher than 4.9 mmol/l were compared for a level of LDL-cholesterol (5.26 (5.12–5.72) mmol/l vs. 5.34 (5.05–5.72) mmol/l in patients from the Tyumen and Kemerovo regions (*p* = 0.680)) and prevalence of statin use (1.6% vs. 4.2%, respectively (*p* = 0.198)).

**Table 1:** Clinical characteristics of participants in the study.

Parameters	Tyumen region n = 131	Kemerovo region n = 186	*p*
Age, years	59 (53–62)	58 (52–61)	**0.020**
Men, n(%)	28 (21.4)	70 (37.6)	**0.003**
CAD, n(%)	16 (12.2)	33 (17.7)	**0.237**
Ischemic stroke, n(%)	1 (0.8)	12 (6.45)	**0.020**

We found 30 patients with definite or probable FH. 16 FH patients were from the Tyumen region and 14 FH patients were from the Kemerovo region. Clinical characteristics of patients with definite or probable FH is provided in Table 2. There were not significant differences in clinical characteristics between FH patients from the Tyumen and Kemerovo regions.

**Table 2:** Clinical characteristics of individuals according to the diagnostic probability of FH.

Parameters	Diagnostic probability of FH			
	Definite or Probable	Possible	Unlikely	All
Number	30	230	2992	3252
Age, years	57 (54–62)*	57 (50–62)	52 (40–59)	52 (41–59)
Men, n(%)	7 (23.3)	73 (31.7)	1086 (36.3)	1166 (35.9)
Xanthomas, n(%)	8 (26.7)	0 (0)	0 (0)	8 (0.2)
Total cholesterol, mmol/l	8.09 (6.94–8.78)	6.83 (5.52–7.33)	5.15 (4.44–5.85)	5.22 (4.47–5.97)
LDL-cholesterol, mmol/l	6.15 (5.19–6.82)	5.07 (3.75–5.46)	3.38 (2.73–4.03)	3.44 (2.77–4.12)
Triglycerides, mmol/l	1.60 (1.13–1.96)	1.44 (1.03–2.02)	1.11 (0.77–1.60)	1.13 (0.80–1.63)
HDL-cholesterol, mmol/l	1.70 (1.52–1.99)	1.65 (1.39–1.89)	1.55 (1.32–1.83)	1.56 (1.33–1.83)
CAD from the ESSE-RF, n(%)	8 (26.7)	23 (10.0)	210 (7.0)	241 (7.4)
CAD after verification, n(%)	12 (40.0)	NA**	NA	NA
Age of CAD starting, years	52 (51–56)	NA	NA	NA
Statins, n(%)	7 (23.3)	20 (8.7)	64 (2.1)	81 (2.5)

*median (25th-75th percentile)

** nonapplicable

Prevalence of tendon xanthomas in the Tyumen and Kemerovo regions was 0.31% (95% CI: 0.01%-0.61%) and 0.19% (95% CI: (-0.05)%-0.43%), respectively (*р* = 0.729). One patient from the Tyumen region had corneal arcus at less than 45 years of age. There were not individuals with corneal arcus at less than 45 years of age in the Kemerovo region.

**Fig 2:**
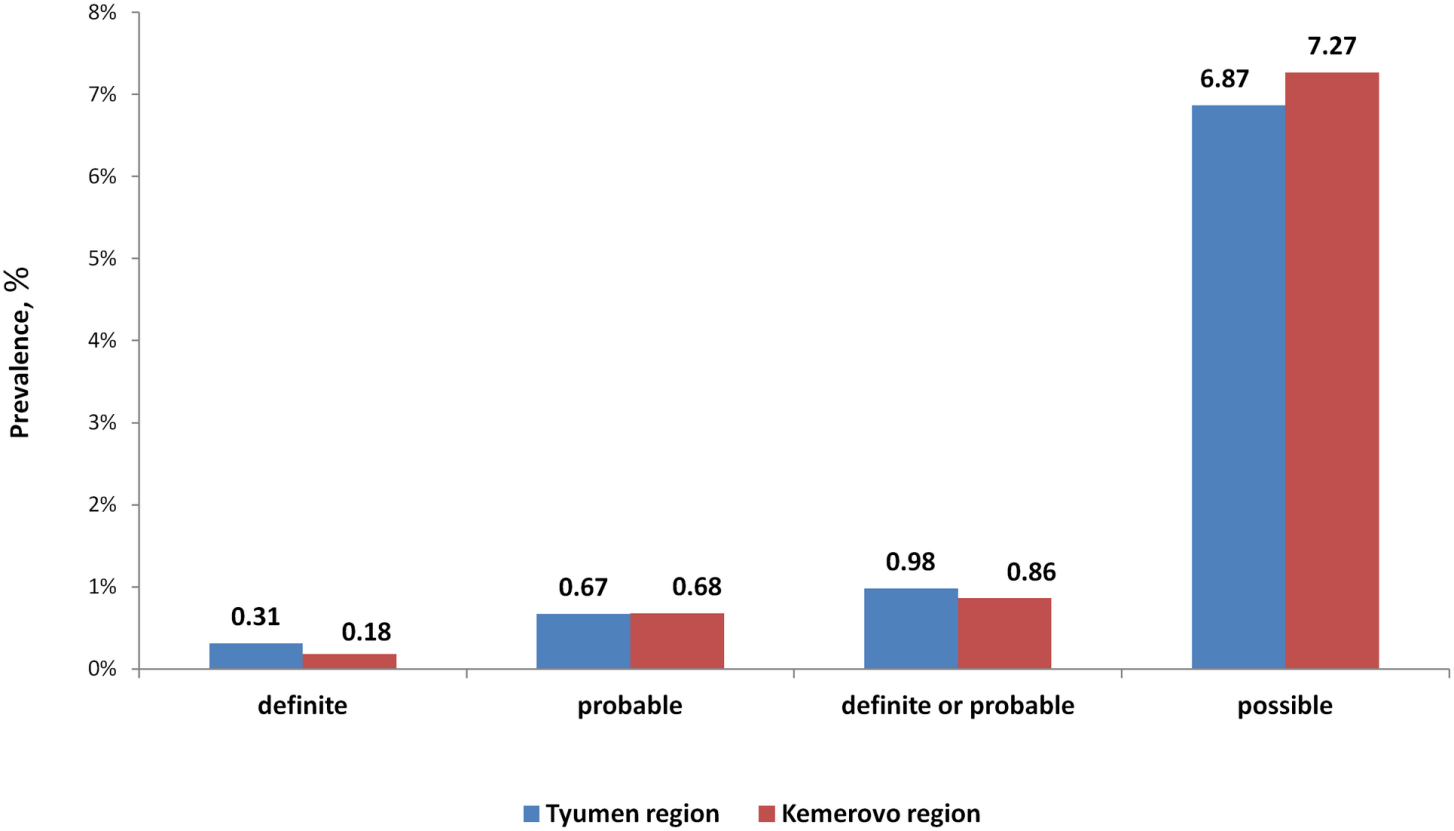
Prevalence of patients with FH in the Tyumen and Kemerovo regions of Russia.

In the Tyumen region, the prevalence of patients classified with definite FH was 0.31% (95% CI: 0.01%-0.61%), probable FH was 0.67% (95% CI: 0.24%-1.10%), definite or probable FH combined was 0.98% (95% CI: 0.47%-1.49%), and possible FH was 6.87% (95% CI: 5.61%-8.13%) (Fig 2).

In the Kemerovo region, the prevalence of patients classified with definite FH was 0.18% (95% CI: (-0.06)%-0.42%), probable FH was 0.68% (0.95% CI: 0.25%-1.11%), definite or probable FH combined was 0.86% (0.95% CI: 0.38%-1.34%), and possible FH was 7.27% (0.95% CI: 5.98%-8.56%). There were not any significant differences in FH prevalence in the regions.

Therefore, in the Russian Federation, the prevalence of patients with definite FH was 0.24% (one in 407) (95% CI: 0.06%-0.42%), probable FH was 0.68% (one in 148) (95% CI: 0.38%-0.98%), definite or probable FH combined was 0.92% (one in 108) (95% CI: 0.58%-1.26%), and possible FH was 7.07% (one in 14) (95% CI: 6.17%-7.97%).

40% (95% CI: 20.8%-59.2%) of patients with definite or probable FH combined in the Tyumen and Kemerovo regions had CAD (Table 2). All detected FH men developed CAD under the age of 55 and all FH women developed CAD under the age of 60. However, only 23% (95% CI: 6.3%-39.7%) of patients with definite or probable FH were on statins. There were no patients with diagnosed definite FH in the Tyumen region that were treated with statins. Among patients with possible FH in both regions, there were 13% and 8.7% of persons who had CAD and who were undergoing treatment with statins, respectively.

**Fig 3:**
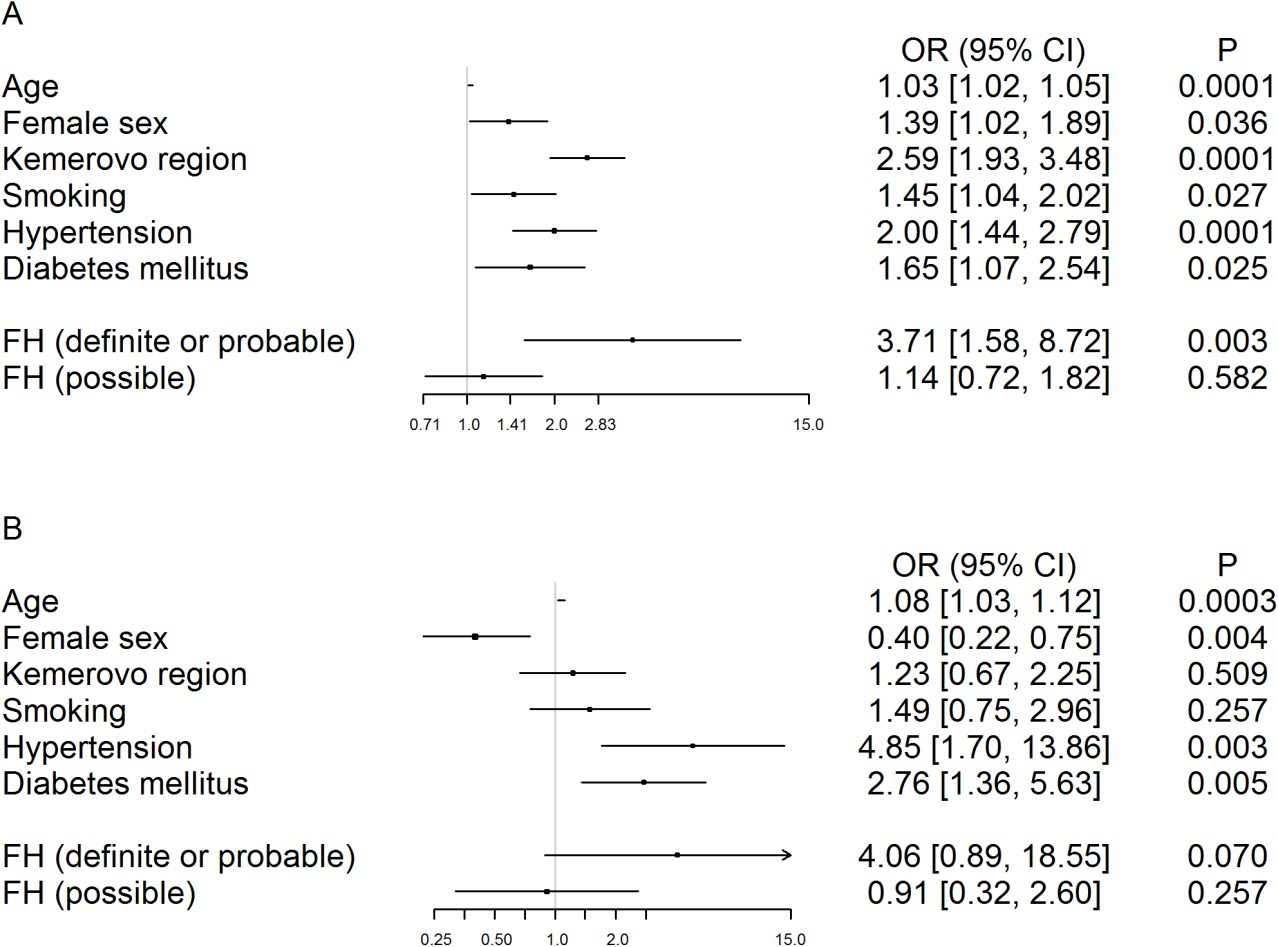
Odds ratio for CAD (A) and MI (B) in patients with FH.

In order to estimate the odds of CAD and MI in persons with FH relative to those with unlikely FH, we took data about CAD and MI diagnosis from the ESSE-RF. We also took the difference in CAD diagnosis in the two regions into account. The adjusted for age, gender, region, smoking, hypertension, and diabetes mellitus, odds ratio for CAD was 3.71 (95% CI: 1.58–8.72) (*p* = 0.003) for individuals with definite or probable FH relative to those who were unlikely to have FH (Fig 3). The adjusted odds ratio for MI was 4.06 (95% CI: 0.89–18.55) (*p* = 0.070) for persons with definite or probable FH relative to those who were unlikely to have FH (Fig 3) The adjusted odds ratio for CAD and MI for patients with possible FH was not significant.

None of the patients with tendon xanthomas had CAD in the Kemerovo region, and only 1 in 5 patients with tendon xanthomas had CAD in the Tyumen region.

## Discussion

Our study is the first to assess the prevalence of FH in Russia. The findings suggest that FH may be present in approximately one in 108 people in Russia. The prevalence of FH found in our study is much more frequent than the theoretical estimated prevalence of one in 500 for heterozygous FH. According to our results, the number of patients with FH in Russia is approximately 1,300,000. However, it might be that only one fifth of those with FH are on cholesterol-lowering treatment.

Advantages of our work are an investigation of the sample from the epidemiological study, face-to-face examination of the participants, and estimation of presence of tendon xanthomata and corneal arcus.

Individuals with LDL-cholesterol less than or equal to 4.9 mmol/l and without statin therapy were not included in the study that can be considered as a limitation of our study. Taking into account that carriers of FH mutations are able to have LDL-cholesterol < 4.9 mmol/l [12], some individuals with FH may not have been detected. In this case, the prevalence of FH in Russia shown in our study is even underestimated.

After The Copenhagen General Population Study, three other population studies were performed. The first one estimated prevalence of FH in the Chinese population [13]. Using modified DLCN definition, which makes a correction for lower levels of LDL-cholesterol in China than in Europe, the prevalence of probable or definite FH was estimated as approximately 1 in 350 (0.28%). It is worth noting that nobody with definite FH was diagnosed. The second study evaluated frequency of FH in the Australian population [14]. The prevalence of patients with definite or probable FH was also 0.28% (94% of those had a probable diagnosis). The third study was the United States National Health and Nutrition Examination Survey, which showed that the FH prevalence in the U.S. was 1 in 250 [15]. It is important to highlight that neither of the listed population studies took into account the presence of tendon xanthomata and corneal arcus in the subjects because they were not recorded.

Thus, all population studies showed us higher prevalence of FH in populations than suggested earlier. The FH frequency is higher in the Russian population than in their foreign counterparts. Maybe, the different results are due to varying approaches to FH diagnostics. FH mutations were genotyped only in individuals from the Danish population while detection of tendon xanthomata and corneal arcus was performed only in our study. An absence of CAD in most of the patients with tendon xanthomata diagnosed in our study emphasizes the significance of physical examination. The much higher prevalence of FH in coronary patients in the Russian Federation as opposed to that in most countries of Europe was also shown in the EUROASPIRE IV [16]. We could suppose that normal levels of cholesterol in Russian adults were higher than those in most European countries. Thus, using the DLCN criteria in Russia might overestimate the prevalence of FH patients, particularly probable FH patients. Unfortunately, the ESSE-RF results with cholesterol levels in Russia have not been published yet, and there is no other Russian epidemiological data about cholesterol, so it is not currently possible to objectively compare the levels of LDL-cholesterol in Russia with those in other countries. As one more possible reason for the high prevalence of FH in the Russian Federation, we should assume that the Russian population differs with regards to genetic profile from other populations. For instance, one study showed that mutations of the LDLR gene in 70% of cases are unique to the Russian population and are not described in other countries [17].

Before the availability of statins, there were several studies reporting the frequency of CAD in FH. In the study of Slack et al [18] the incidence of CAD by 50 years of age in FH men and women was 85.4% and 56.5%, respectively. In the work of Stone et al [18], where analysis of cardiovascular status in adult relatives of kindred affected with familial hyperbetalipoproteinemia was carried out, the cumulative probability of nonfatal or fatal CAD by age 60 was 52% and 32.8% of FH men and women respectively. In our study, only 23% of FH patients were treated with statins, therefore we received data on CAD prevalence in FH patients close to that found in treatment-naive FH patients. Prevalence of CAD in FH patients in our study was 40%. It is consistent with the above study of Stone et al [19] and other reports of partially treated individuals. In the study describing FH in China, 43.2% of FH patients had CAD, while 16% of them were on cholesterol-lowering therapy [13].

In our study, compared with non-FH subjects, patients with FH had 3.7-fold odds of CAD. Taking into consideration the fact that the most evidence of CAD risk in FH is obtained from registers and lipid clinics, where, as a result, patients may have a more severe form of FH, it is suitable to match our results with those of other population studies, which give us a more accurate idea of the natural history of the disease. According to the Copenhagen General Population Study, CAD and MI risks in FH are respectively 13 and 10 times higher in individuals with FH than in the general population with regards to being off and on cholesterol-lowering therapy [6]. FH increased the risk of CAD about 20 times in the Australian and Chinese populations [13, 14]. Risk was calculated in treatment-naive patients only in the above study. Therefore, we found that CAD risk in FH is significantly higher than average in the Russian population, but it is lower than averages found in various other FH populations. We suggest that these differences are due to varying methodologies of CAD diagnostics. In the ESSE-RF, an epidemiologic approach to CAD diagnostics was used—in particular, the majority of participants were diagnosed with CAD by means of the Rose Angina Questionnaire, which is known to be less informative regarding women [20], who formed the main part of the study sample. Perhaps, overdiagnosis of CAD in the ESSE-RF induced lower CAD risk of FH in our study.

Considering the high FH prevalence in Russia and the fact that everybody with FH was newly diagnosed in our study, we conclude that FH is underdiagnosed in Russia. Regarding the high prevalence of CAD in individuals with FH and the very low percentage of FH patients treated with statins, we can also deduce that FH is undertreated in Russia.

## Conclusions

The prevalence of FH in Russia may be significantly higher than estimated. According to our results, the prevalence of FH in the West Siberian region of the Russian Federation is one in 108. Almost half of individuals with FH have CAD, additionally, the majority of patients have early onset of CAD. However, only one fifth of those with FH undergo cholesterol-lowering treatment. Underdiagnosis and undertreatment of FH in Russia underline the need for the intensification of FH detection with early and aggressive cholesterol-lowering treatment.
